# Therapeutic applications of gut microbes in cardiometabolic diseases: current state and perspectives

**DOI:** 10.1007/s00253-024-13007-7

**Published:** 2024-01-20

**Authors:** Lin Yuan, Ying Li, Moutong Chen, Liang Xue, Juan Wang, Yu Ding, Qihui Gu, Jumei Zhang, Hui Zhao, Xinqiang Xie, Qingping Wu

**Affiliations:** 1 School of Food and Biological Engineering, Shaanxi University of Science and Technology, Xi’an, 710021 China; 2 Guangdong Provincial Key Laboratory of Microbial Safety and Health, State Key Laboratory of Applied Microbiology Southern China, Institute of Microbiology, Key Laboratory of Agricultural Microbiomics and Precision Application, Ministry of Agriculture and Rural Affairs, Guangdong Academy of Sciences, Guangzhou, 510070 China; 3 College of Food Science, South China Agricultural University, Guangzhou, 510642 China; 4 Department of Food Science and Engineering, Institute of Food Safety and Nutrition, College of Science & Engineering, Jinan University, Guangzhou, 510632 China

**Keywords:** Cardiometabolic disease, Gut microbiome, Dietary nutrition

## Abstract

**Abstract:**

Cardiometabolic disease (CMD) encompasses a range of diseases such as hypertension, atherosclerosis, heart failure, obesity, and type 2 diabetes. Recent findings about CMD’s interaction with gut microbiota have broadened our understanding of how diet and nutrition drive microbes to influence CMD. However, the translation of basic research into the clinic has not been smooth, and dietary nutrition and probiotic supplementation have yet to show significant evidence of the therapeutic benefits of CMD. In addition, the published reviews do not suggest the core microbiota or metabolite classes that influence CMD, and systematically elucidate the causal relationship between host disease phenotypes-microbiome. The aim of this review is to highlight the complex interaction of the gut microbiota and their metabolites with CMD progression and to further centralize and conceptualize the mechanisms of action between microbial and host disease phenotypes. We also discuss the potential of targeting modulations of gut microbes and metabolites as new targets for prevention and treatment of CMD, including the use of emerging technologies such as fecal microbiota transplantation and nanomedicine.

**Key points:**

• *To highlight the complex interaction of the gut microbiota and their metabolites with CMD progression and to further centralize and conceptualize the mechanisms of action between microbial and host disease phenotypes*.

• *We also discuss the potential of targeting modulations of gut microbes and metabolites as new targets for prevention and treatment of CMD, including the use of emerging technologies such as FMT and nanomedicine*.

• *Our study provides insight into identification-specific microbiomes and metabolites involved in CMD, and microbial-host changes and physiological factors as disease phenotypes develop, which will help to map the microbiome individually and capture pathogenic mechanisms as a whole*.

**Graphical Abstract:**

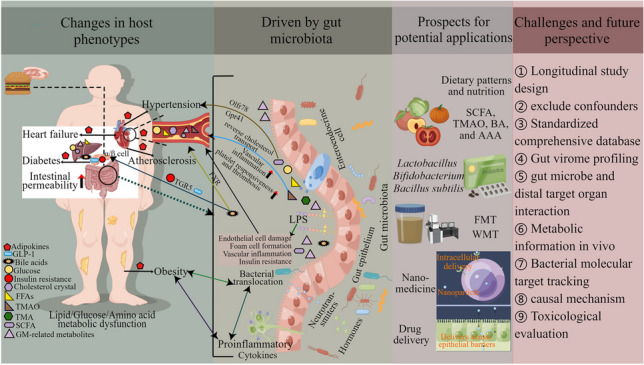

**Supplementary Information:**

The online version contains supplementary material available at 10.1007/s00253-024-13007-7.

## Introduction

Cardiometabolic disease (CMD), such as atherosclerosis, heart failure, hypertension, and hyperlipidemia (Tsao et al. 2022), is highly prevalent. The prevalence rate has been rising steadily for decades, becoming the main cause of death in the global population (Roth et al. 2020). Although the recommended treatment methods, including high-dose statins and lifestyle risk factors, were consistently used, the efficacy was not ideal, and adverse reactions still existed. The cause of CMD is still under study, but it is believed to be influenced by genetic and environmental factors. The risk factors associated with CMD can be unchangeable (genetic) or changeable (such as drinking, smoking, diet, and exercise). In conclusion, these observations indicated that many pathophysiological mechanisms of CMD had not been fully understood, and convincing data from clinical research, biochemistry, bioinformatics, pharmacology, and basic medicine would enhance our understanding of cardiovascular diseases (CMD) and the feasibility of prevention.

The human gastrointestinal tract is composed of trillions of microorganisms, including beneficial or pathogenic bacteria, fungi, and viruses. These microorganisms and their genomes are collectively referred to as intestinal flora (Loscalzo 2013). The intestinal flora and the metabolic function of the host interact, such as the decomposition and absorption of nutrients and the regulation of immune function, are essential to maintain the intestinal and external balance (Clemente et al. 2012; Loscalzo 2013). Recent studies have revealed the potential role of intestinal microbiome in CMD disease. And there is mounting evidence that changes in intestinal microbiota and its related metabolites may affect the initiation and progression of CMD, such as atherosclerosis, heart failure, and hypertension (Ascher and Reinhardt 2018; Koeth et al. 2013). The treatment of disease phenotype through fecal flora transplantation has been applied, which shows that the intestinal flora is sufficient to face the occurrence of CMD and atherosclerosis (Bäckhed et al. 2004; Ridaura et al. 2013), and also sufficient to transmit environmental protective role. The intestinal microbiome is also characterized as an endocrine organ that affects metabolism. However, this host-centric view largely ignores the complex nature of the intestinal microbiota.

In recent years, several study authors have summarized the role of gut microbiota in cardiometabolic disease (CMD) and the possible mechanisms by which probiotics intervene in cardiometabolic disease. These review articles provide theoretical basis for the intervention of CMD by targeting intestinal flora. However, these reviews did not suggest a core class of microbiota or metabolites that influence CMD, elucidate the causal relationship between host disease phenotypes-microbiome, or the possible application of personalized microbiome therapy for CMD. The aim of this review is to highlight the complex interaction of the gut microbiota and their metabolites with CMD progression and to further centralize and conceptualize the mechanisms of action between microbial and host disease phenotypes. We also discuss the potential of targeting modulations of gut microbes and metabolites as new targets for prevention and treatment of CMD, including the use of emerging technologies such as FMT and nanomedicine. This review is focused on three main key concepts. Firstly, there is increasing evidence that gut microbiota affects host physiological function through multiple pathways and specific receptors, and plays an important role in the pathology of host CMD. Thus, we describe the potential role of gut microbiota influencing CMD and the gut-X axis. Secondly, contributing roles of gut microbiota metabolites or secondary metabolites has been observed in attenuating CMD via neuroimmune pathways, as an immunomodulator or endocrine promoter. Thirdly, the rapid development of multi-omics techniques helps us to identify specific microbiomes and metabolites involved in CMD and to gain insight into microbial-host changes as disease phenotypes develop, which facilitates the development of live bacteria drugs and lead compounds, thus providing promising new avenues for therapeutic options. In this process, we identified knowledge gaps and limitations in current approaches to inform the development of drugs that regulate gut microbiota as new therapeutic targets for CMD.

## Role of gut microbiota in host physiology

The human microbiome includes bacteria, viruses, single-celled eukaryotes, and archaea. And the microbes that live in symbiosis with the host are called microbiota, and their genome is called the “microbiome.” *Lactobacillus*, *bifidobacteria*, and other coliforms have been demonstrated as the most promising probiotics and their role in the prevention of degenerative diseases such as obesity, diabetes, cancer, cardiovascular disease, malignancies, liver disease, and inflammatory bowel disease (Azad et al. 2018). Intestinal microorganisms can also interact with elements of the host neuroendocrine system to modify behaviors relevant to social behavior, eating behavior, cognition, addiction, and sexual behavior (Cussotto et al. 2018). It is worth pointing out that gut microbes play an important role in mediating hormone release and thus regulating host metabolism. Gut microbes and metabolites influence the release of a variety of hormones by intestinal endocrine cells within the lining of the intestinal mucosa, including peptide YY, gastric inhibitory polypeptide, cholecystokinin, Glucagon-like peptide-1 (GLP-1), and 5-hydroxytryptamine (5-HT), which play a role in regulating key metabolic processes such as insulin sensitivity, fat storage, glucose tolerance, and appetite (Martin et al. 2019). In conclusion, gut microbiota can significantly affect host immunity and metabolism, and play an important role in maintaining the overall health of the host and resisting disease.

## Intestinal leakage causes the entry of intestinal microbial derivatives and inflammation

Intestinal epithelial cells and the mucus they produce, as well as mucous membranes, form the intestinal barrier. The intestinal barrier prevents the invasion of pathogenic antigens. Impairment of intestinal wall and barrier function is often observed in patients with heart failure (Tang et al. 2019a). The increase of inflammatory factors in blood circulation is related to the severity of heart failure (Munger et al. 1996; Rauchhaus et al. 2000). Lipopolysaccharides produced by gram-negative bacteria in the gut can enter the host circulation through the impaired intestinal barrier. TLR (Toll-like receptor) on the surface of immune cells recognizes lipopolysaccharide and releases pro-inflammatory cytokines that put the host in a pro-inflammatory state (Hug et al. 2018). Some studies have shown that after *Akkermania* treatment, the level of lipopolysaccharide (LPS) in plasma of patients with metabolic syndrome (MS) is reduced (Depommier et al. 2019). Intestinal leakage is not the only cause of CMD. Many diseases are also associated with intestinal leakage. The relationship between CMD susceptibility and intestinal barrier damage changes in gut flora and metabolites, and host inflammatory response needs further exploration. Identifying the mechanism by which gut flora participates in the progress of CMD by triggering systemic immune response through the change of intestinal barrier will help to provide a new therapeutic approach.

## Gut microbiome in CMD: altered composition of gut microbiota

**Fig. 1:**
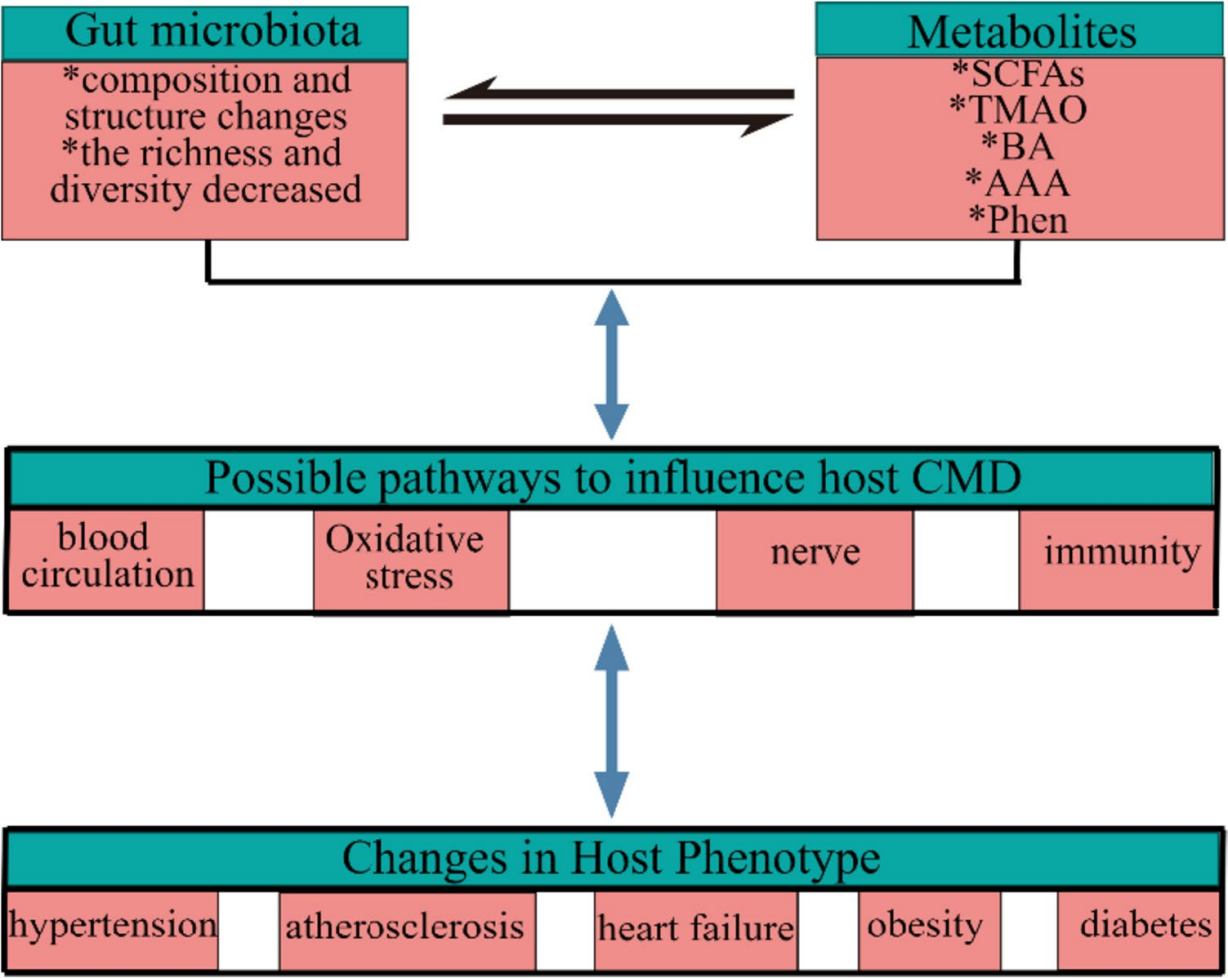
The changes of gut microbiota and its metabolites lead to CMD in the host. SCFA, short-chain fatty acid; TMAO, trimethylamine oxide; BA, bile acids; AAA, aromatic amino acids; Phen, phenylacetylglutamine; CMD, cardiometabolic disease

An imbalance in the gut microbiome is called dysbiosis. The correlation between gut microbiota dysbiosis and many diseases/phenotypes has been a hot topic in the past decade. Identifying specific microbiomes associated with disease susceptibility is a fascinating research. Most gut microbial communities are composed of *Bacteroides*, *Proteobacteria*, *Actinobacteria*, *Firmicutes*, and *Cerrucomicrobia*, with *Bacteroides* and *Firmicutes* as the main phylum, and the ratio of *Bacteroide*s to *Firmicutes* is also considered to be associated with the health (Tang et al. 2019b). However, there are differences among different individuals, different diseases, and different intestinal sites. The change of bacterial diversity is also associated with host genome and environmental factors. However, with the rapid development of sequencing technology, these microorganisms can be identified and characterized. It is worth mentioning that we need to identify a core group of bacteria and further study the pathogenesis of cardiovascular metabolic disorders from the perspective of bacterial functional genomics. Common diseases in which gut microbiota and their metabolites lead to CMD in the host through different pathways are shown in Fig. 1 (by Figdraw).

### Hypertension

Hypertension is a risk factor on cardiovascular disease (Qato et al. 2018). The research in the experimental animal model showed that the regulation of gut flora was helpful to prevent hypertension (Jama et al. 2019b). Most studies have found that the gut microbiota of patients with hypertension was identified a reduced alpha diversity (De la Cuesta-Zuluaga et al. 2018; Jackson et al. 2018; Sun et al. 2019; Verhaar et al. 2020). Recent research by Yuan et al. also showed that *Christensenellaceae* and *Streptococcaceae* were the dominant microbes in the hypertensive animals (Yuan et al. 2022). A large population cohort study analyzed the relationship between gut microbiota and blood pressure (BP) in 6953 Finns aged 25 to 74*.* There was a negative correlation between 19 different *lactobacilli* and BP index (Palmu et al. 2020). Losartan, a drug used to treat hypertension, was believed to attenuate the gut dysbiosis, improve the integrity of the intestinal tract, thus partially helping to protect the vascular system and lower BP (Robles-Vera et al. 2020).

**Fig. 2:**
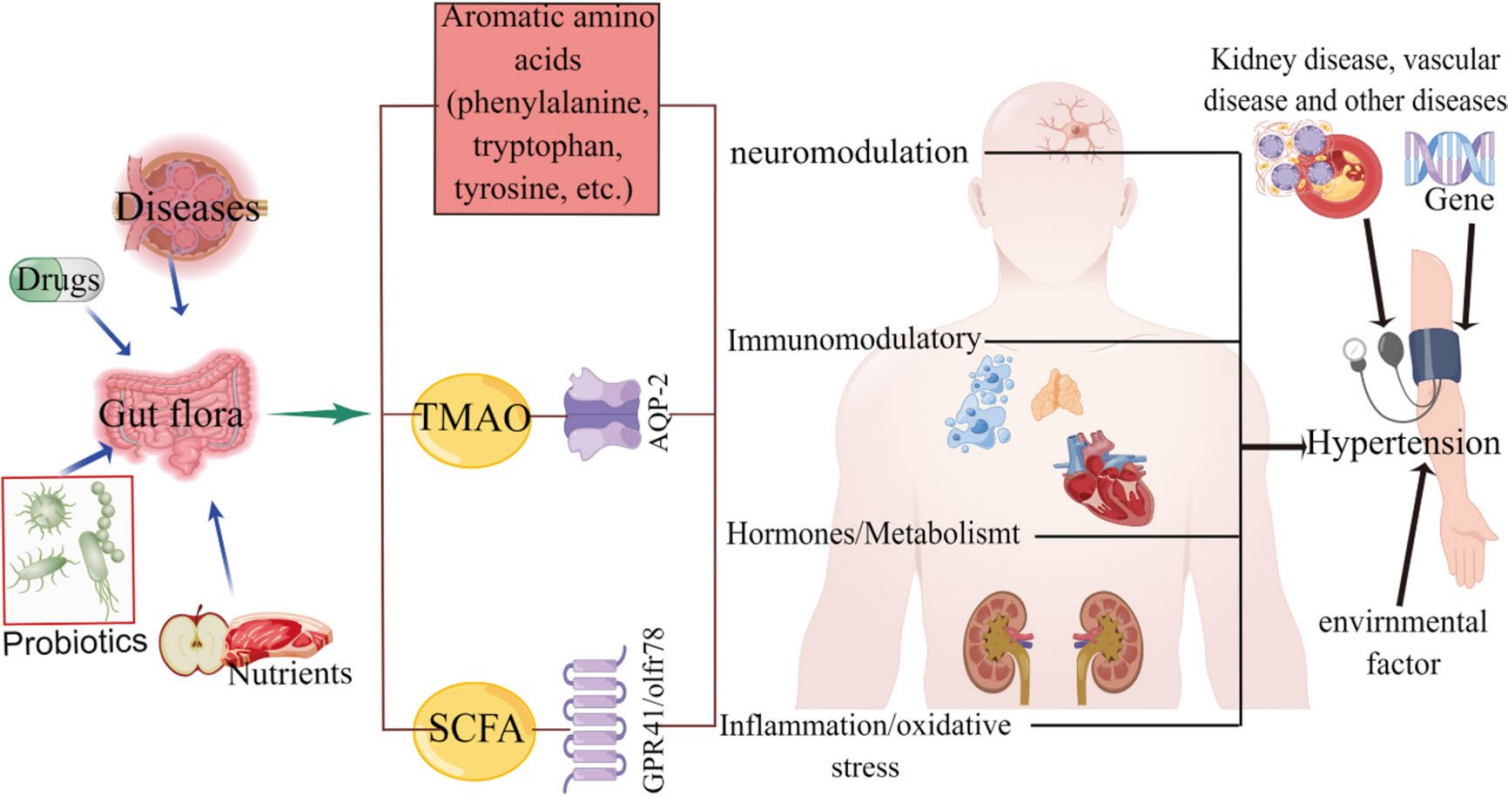
Effect of gut microbiota on hypertension. SCFA, short-chain fatty acid, TMAO, trimethylamine oxide

The absence of short-chain fatty acid (SCFA)–producing bacteria has been reported as a characteristic of gut flora in patients with hypertension (Calderon-Perez et al. 2020; Jama et al. 2019a). SCFAs can be activated by receptors to regulate renin secretion and achieve the purpose of BP regulation. The intestinal metabolite TMAO may also be related to the occurrence of hypertension. The specific mechanism may be related to more water reabsorption caused by aquaporin-2 (AQP-2) (Min et al. 2018). The possible pathways by which the gut microbiota may influence hypertension are illustrated in Fig. 2 (by Figdraw). A recent study has shown that the high-salt diet significantly changed the gut flora composition of rats, decreased *bacteroidetes frailty* and intestinal arachidonic acid levels, and increased the serum and intestinal corticosterone levels (Yan et al. 2020). Moreover, some aromatic amino acids (phenylalanine, tryptophan, tyrosine) metabolized in the intestines can also affect hypertension (Jin et al. 2021; Wang et al. 2021c; Yuan et al. 2022). Interestingly, fecal microbiome composition is associated with BP, but there are racial and gender differences (Verhaar et al. 2020). Alterations in the gut microbiota associated with hypertension are shown in Table S1.

### Atherosclerosis

Patients with atherosclerosis have the accumulation of low-density lipoprotein plaque buildup and vascular cell dysfunction in their blood vessels (Kazemian et al. 2020). Chronic inflammation is a potential driving factor for atherosclerosis. Annika et al. (Jonsson and Bäckhed 2017) summarized three factors affecting atherosclerosis by gut flora, including harmful inflammatory reaction, metabolism of cholesterol and lipids by gut flora, and dietary and specific components of gut flora metabolism. An accumulating amount of research demonstrated that atherosclerosis is associated with specific bacterial groups, but its specific causal mechanism and downstream molecular pathways need to be further explored. Probiotics and prebiotics can regulate gut flora to improve cardiovascular metabolic diseases (Ma and Li 2018). A recent study showed that consumption of Brussels chicory increased the stability of atherosclerotic plaque in mice. Its mechanism could be associated with the reduction of intestinal permeability, the concentration of LPS in feces and serum, the promotion of plaque macrophage polarization to M2-like phenotype, and the change of gut microbial composition (Li et al. 2022c). The regulation of gut microbiota in this study could be mediated by dietary fiber and inulin in Brussels chicory (Johnson and Weir 2022). However, not all studies on inulin regulating intestinal flora have shown antiatherogenic effect (Hoving et al. 2018). The exact pathophysiological mechanism of atherosclerosis formation and its causal relationship with changes in gut microbiota deserve attention. Finally, we summarized changes in gut microbiota in patients and animals with atherosclerosis from previous studies, as shown in Table S2.

### Heart failure

Heart failure (HF) is a disease in which the heart’s ability to pump blood decreases (Jin et al. 2021). HF could lead to splanchnic circulation congestion and impaired intestinal barrier function, thus increasing the bacterial products in the blood circulation of the system and heightening the inflammatory state (Tang et al. 2019b). The diversity of gut microbiota was significantly decreased in HF patients, and the key intestinal bacteria were reduced (Luedde et al. 2017). Another study also showed that the gut flora richness of chronic HF was low, and butyrate-producing bacteria were significantly reduced (Kummen et al. 2018). A two-sample Mendelian randomized study showed that the gene prediction results indicated that candida, campylobacter, and shigella in the gut flora were not associated with the incidence rate of HF (Luo et al. 2022).

A disturbed microflora of the gut may affect the progress of heart failure through the interaction between the intestinal barrier damage, immune disorder, and neurohormone release. In addition, the intestinal disorder will also affect atherosclerosis, hypertension, diabetes, and obesity, which in turn aggravate the damage of gut barrier (Sata et al. 2020). Heart failure reduces patients’ quality of life and causes premature death. Lifestyle intervention is considered to be an effective way to improve the prognosis and quality of life of patients with heart failure. High levels of serum alpha-linolenic acid were associated with better follow-up outcomes in patients with heart failure, and dietary interventions rich in alpha-linolenic acid could help prevent and treat heart failure, according to a new study published recently (Lázaro et al. 2022).

### Obesity and type 2 diabetes mellitus

Obesity has been on the rise over the past few decades, posing a threat to public health. Although obesity itself is not harmful, it creates conditions for the development of MS and cardiovascular disease (CVD). Modern diets and sedentary lifestyles are thought to be the main reasons for the rise in obesity, but they do not fully explain mechanisms contributing to obesity.

An increasing body of evidence suggests that gut flora is associated with the development of obesity and associated metabolic diseases (Torres-Fuentes et al. 2017; Zhu et al. 2022). Gut flora is closely associated to host food digestion, nutrient absorption, energy metabolism, and central appetite, which are all associated with obesity (Wijdeveld et al. 2020). *Bifidobacterium longum* and *Parabacteroides goldsteinii* have been reported to reduce body weight and regulate gut flora in HFD-fed mice, and also reducing fat accumulation, reducing insulin resistance, and increasing glucose tolerance (Schellekens et al. 2021; Wu et al. 2019). In addition, obesity may also be associated with the *Firmitutes/Bacteroidetes* ratio, *Akkermania*, *Bifidobasteria*, and *Enteractor* (Lee et al. 2019). However, it is interesting to note that the *Firmicutes*/*Bacteroidetes* ratio still needs further confirmation in distinguishing obesity from normal gut microbiota (Moran-Ramos et al. 2017).

Several studies have shown that the regulation of gut flora to attenuate obesity and dyslipidemia may be associated with the regulation of intestinal metabolites such as branched amino acid and SCFAs (Li et al. 2020b; Zeng et al. 2020). Besides, the fecal microbiota of severely obese patients decreased, and the biotin synthesis and transport potential of the flora decreased, which was associated with the host metabolism and inflammatory phenotype (Belda et al. 2022). The effect of microbiota on obesity is also considered to be associated with endotoxemia, insulin resistance, and intestinal permeability (Aydin et al. 2018; Cornejo-Pareja et al. 2019). Gut flora plays a role in mediating inflammation and insulin resistance. Multi‑omics data analysis of healthy individuals and prediabetes individuals showed that there was a specific host-microbial interaction between insulin resistance and insulin-sensitive individuals (Zhou et al. 2019).

The reduction of butyrate-producing bacteria could lead to the multiplication of pathogens, which indicated that butyrate-producing bacteria were significantly important for the stability of the intestinal system. When butyrate production decreased, the gut flora function of diabetes mellitus type 2 (T2DM) patients could be enriched in the process of branched chain amino acid production, lactate, monosaccharide, and B group vitamins metabolism (Alvarez-Silva et al. 2021; Belda et al. 2022; Thingholm et al. 2019; Wu et al. 2020). Branchchain amino acids have also been associated with cardiometabolic disease, and *Prevotella copri* has been found to increase the level of Branchchain amino acids in the peripheral circulation of animals causing glucose intolerance (Arany and Neinast 2018; Caussy and Loomba 2018; Pedersen et al. 2016; White and Newgard 2019; Yang et al. 2021a).

**Fig. 3:**
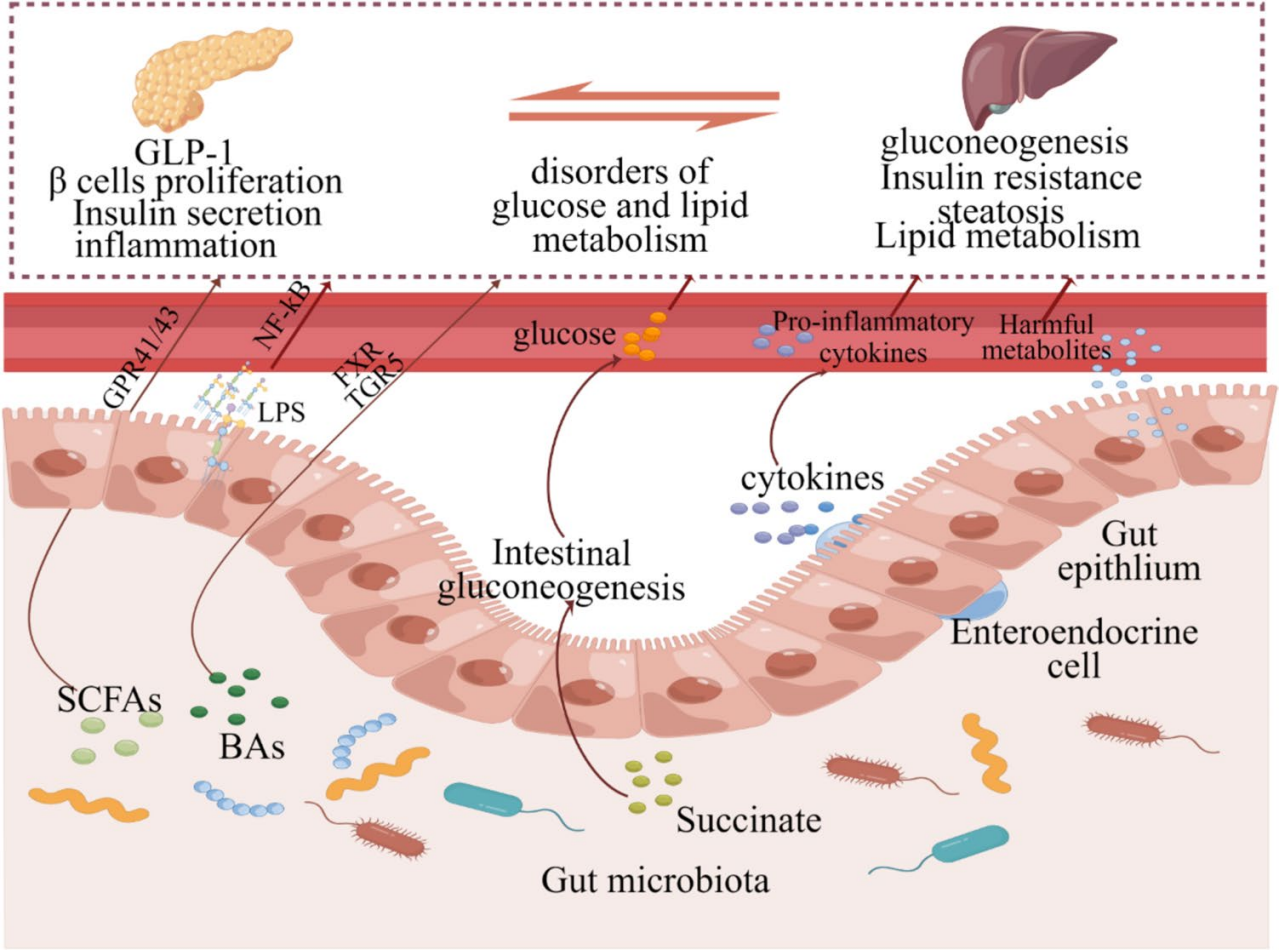
The gut microbiota is involved in the development of type 2 diabetes through multiple molecular mechanisms, such as damage to the gut barrier, induction of insulin inflammation, and liver gluconeogenesis. SCFA, short-chain fatty acid; BA, bile acid; LPS, lipopolysaccharide; FXR, Farnesoid X receptor; TGR5, Takeda G protein–coupled receptor 5; GPR, G protein–coupled receptor; NF-κB, nuclear transcription factor-κB

The underlying systematic mechanism between gut microbiota and type 2 diabetes is described in Fig. 3 (De Vadder et al. 2016; Guo et al. 2022; Jiang et al. 2022; Yang et al. 2021a; Zhao et al. 2022) (by Figdraw). Despite the diversity of gut flora, individuals with obesity, insulin resistance, and dyslipidemia often exhibit low gut bacterial richness (Sun et al. 2018). Gut microbiota is a promising target for the treatment of metabolic diseases such as obesity and diabetes; however, more clinical and basic research is needed to elucidate the exact mechanism. The development of drugs based on precise gene modification of microbiome may be an important direction for the prevention and treatment of T2DM (Liu and Lou 2020). In addition, there are many indicators in the clinical diagnosis of diabetes. In the future, more attention should be paid to the relationship between gut microbiota and the degree of insulin resistance, diabetes classification and obesity classification, and other complications.

## Changes in microbial metabolites

### SCFA

SCFAs can be produced by the host or by intestinal microorganisms that ferment dietary fiber such as hemicelluloses, cellulose, fructan, and resistant starch, usually containing five or fewer carbons (Bose et al. 2019; Deehan et al. 2020). In the colon, SCFAs were mainly composed of acetic acid, propionic acid, and butyric acid, with the proportion of 60:20:20. The relationship between SCFAs and glucose and lipid metabolism, appetite regulation and intestinal transit time has been confirmed in animal models (Chakaroun et al. 2022; Koh and Backhed 2020). Reduction of intestinal butyrate is generally observed in cardiometabolic diseases and CVD, and is associated with disease severity (Chakaroun et al. 2022).

**Fig. 4:**
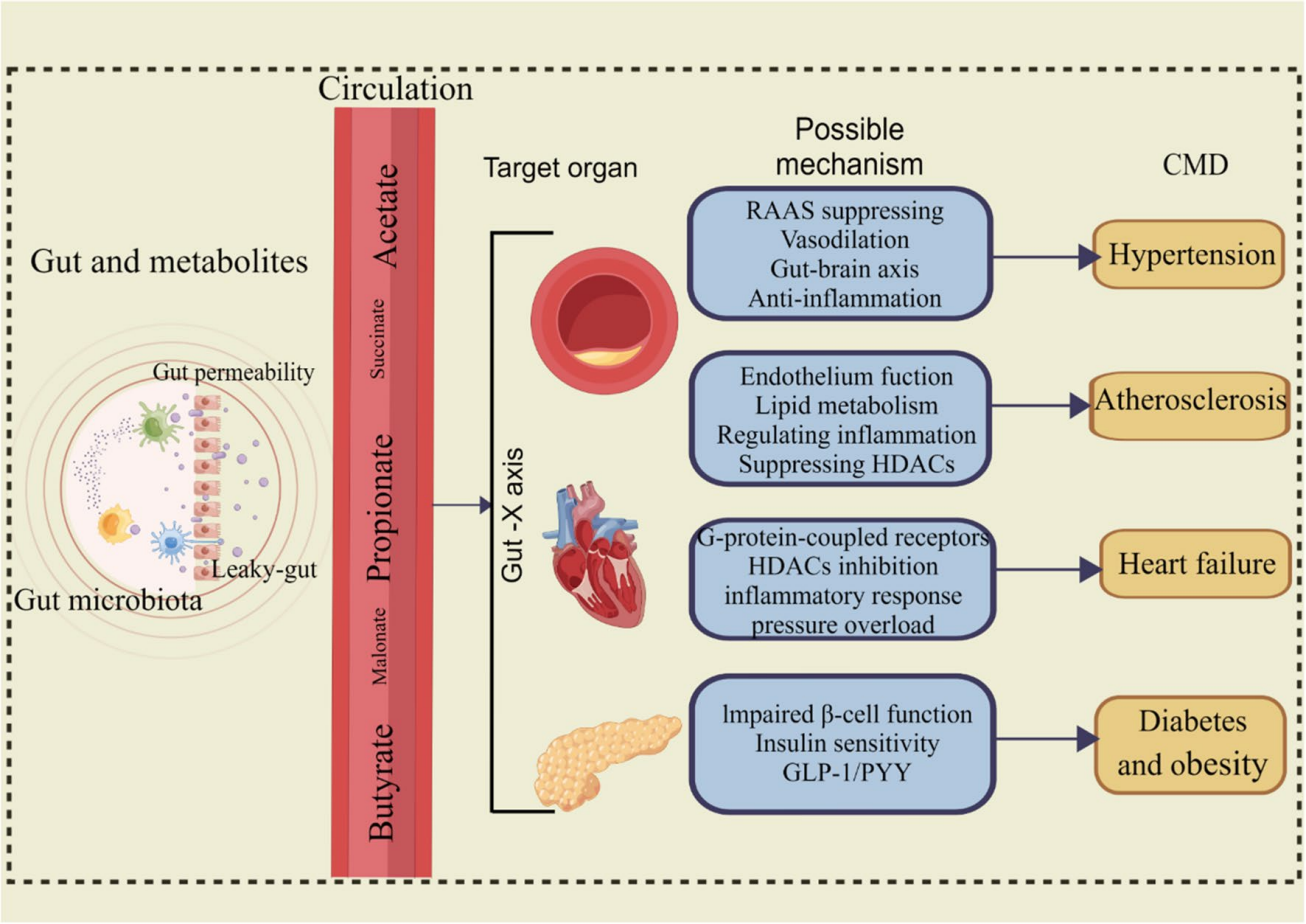
The short-chain fatty acid (SCFA) is involved in the development of cardiometabolic disease (CMD) through multiple molecular mechanisms, such as anti-inflammation, lipid metabolism, and insulin sensitivity. RAAS, renin angiotensin aldosterone system; HDAC, histone deacetylase; GLP-1, Glucagon-like peptide-1; PYY, peptide YY

The number of SCFA-producing bacteria in essential hypertension patients was significantly lower than that in healthy controls (Liu et al. 2021b). However, it must be noted that a longitudinal cohort study with an average follow-up of 5 years showed that fecal acetate, propionate, and butyrate were all higher in hypertensive patients than in the normal population, possibly due to higher excretion of SCFA (Huart et al. 2021). A recent study showed that maternal 3, 3-dimethyl-1-butano could increase the plasma acetic acid, reduce the expression of GPR43 and GPR109A, and thus help to favor vasodilatation and lower BP (Hsu et al. 2021). SCFA in patients with acute myocardial infarction was significantly lower than that in healthy controls, indicating that it may be a marker for the diagnosis of transient myocardial infarction (Guo et al. 2021). Propionic acid has parasympathetic excitatory effect. Zhou et al. studied the nutritional intervention effect of propionic acid on the heart after myocardial infarction. The results showed that oral propionic acid might partially activate the parasympathetic nerve based on the gut-brain axis and improve ventricular electrical remodeling in a rat model with myocardial infarction (Zhou et al. 2021). However, a study of 441 adults living in the community found that the high concentration of SCFA in feces was negatively associated with the diversity of microbiota, and was associated with the markers of intestinal permeability, obesity, hypertension, and metabolic disorder. The authors believed that the association between high fecal SCFAs and obesity and heart metabolic disorder might be due to the low absorption efficiency of SCFA (De la Cuesta-Zuluaga et al. 2018). Yves A et al. have found that the absolute and relative increase of propionic acid in the cecum is associated with the alleviation of atherosclerosis, liver steatosis, and inflammation (Millet et al. 2022). After major intestinal cholesterol transporter Niemann-Pick C1-like protein 1 was inhibited by propionic acid, the levels of total cholesterol and low-density lipoprotein decreased in atherosclerosis model mice, attenuating atherosclerosis (Haghikia et al. 2022). Conversely, it is interesting that succinate may activate hypoxia-inducible factor 1-alpha, nuclear factor kappa beta, and renin angiotensin system to make vascular fibrosis and smooth muscle cell proliferation and migration; it can also bind GPR91 receptor to activate and mediate macrophage polarization and cause endothelial dysfunction (Hu et al. 2022a; Zhang et al. 2022). The study of Sarah K. Kirschner et al. showed that the plasma concentrations of propionate, isovalerate, and butyrate in patients with heart failure were significantly lower than those in healthy controls, while the concentrations of acetate and valerate were not different (Kirschner et al. 2022). SCFA also has effects on neuroimmunity and endocrine function. For example, when the concentration of propionic acid and butyric acid in the body of patients with congestive heart failure is high, heart function and leg muscles tend to be healthier (Dalile et al. 2019; Frampton et al. 2020). Furthermore, intestinal-derived SCFAs can activate its receptors GPR43 and GPR41 and control obesity by participating in fat accumulation and inhibiting lipolysis in adipocytes (Sun et al. 2018). The underlying mechanism between SCFAs and CMD is described in Fig. 4 (Alhabeeb et al. 2021; Chakaroun et al. 2023; Chen et al. 2020b; Hu et al. 2022b; Lu et al. 2022; Pakhomov and Baugh 2021; Yao et al. 2020; Yoo et al. 2022; Yukino-Iwashita et al. 2022) (by Figdraw).

SCFA is usually regarded as the terminal product of microbial catabolism, representing the readout of saccharic metabolism of the entire microbial community, and plays an important role in various cardiovascular diseases. Hypertension, atherosclerosis, heart failure, and diabetes are all accompanied by changes in SCFA and SCFA-producing bacteria, which may be a new biomarker for risk prediction of CMD. The functions of SCFA in metabolism, neuroimmunity, and anti-inflammation play a certain role in cardiovascular homeostasis. Reducing the risk of cardiovascular disease by supplementing specific fiber diets or directly supplementing SCFA is considered promising. However, the role of single SCFAs or combined SCFAs in the pathophysiology of different cells and diseases needs further study.

### TMAO

TMA produced from choline, betaine, and L-carnitine metabolized by intestinal flora becomes TMAO after oxidation. TMAO is an intestinal metabolite, and its potential role in CVD has been confirmed. TMAO level is associated with promoting atherosclerosis and a moderately increased risk of CVD. TMAO is also involved in such as platelet activation, endothelial dysfunction, and thrombosis (Zhu et al. 2020). TMAO can regulate the expression of SERPINE1 to promote blood coagulation, which can accelerate the formation of atherosclerosis (Díez-Ricote et al. 2022). Recent studies have shown that TMAO increased inflammatory factors such as intercellular adhesion molecule-1 (ICAM1) and interleukin-6 (IL-6), oxidized low-density lipoprotein, and induced CD36 expression, thus aggravating atherosclerosis, while MAPK/JNK inhibitors could reduce TMAO-induced CD36 expression (Catar et al. 2022; Geng et al. 2018; Huang et al. 2022a).

The level of TMAO in patients with heart failure is closely associated with adverse events (mortality and/or rehospitalisation) (Suzuki et al. 2019). Multiple population cohort prospective studies have shown that TMAO and its precursors such as carnitine are predictors of adverse cardiovascular events in patients with heart failure (Israr et al. 2021; Suzuki et al. 2019; Wei et al. 2022; Zhou et al. 2020). Furthermore, a large number of latest lines of evidence indicated that the high level of TMAO in blood circulation was also associated with the progression of hypertension and related CVD (Huang et al. 2022b; Li et al. 2022a; Yang et al. 2022). A study suggested that TMAO might be a target for berberine to improve hypertension and complications (Wang et al. 2022). Hypertension and circulating TMAO concentration demonstrated significant positive dose dependence (Ge et al. 2020). However, a study on the relationship between TMAO and hypertension showed that high total choline intake would increase the level of TMAO, and there was a significant negative correlation between total choline and BP within a certain range (He et al. 2022). The relationship between TMAO and hypertension has not been elucidated, and the involvement of TMAO in the pathophysiological process of hypertension needs to be further discovered. Another prospective cohort study (2088 Chinese community participants) showed that serum TMAO levels were positively associated with the risk of type 2 diabetes and elevated fasting blood glucose in middle-aged and elderly Chinese (Li et al. 2022d).

In addition, TMAO, a metabolite derived from gut microbiota, could induce cardiac hypertrophy and fibrosis, which involved Smad3 signal transduction (Li et al. 2019). A prospective population study by Tang et al. showed that in apparently healthy individuals, the risk of CVD can be predicted by the increase of plasma TMAO level (Tang et al. 2021). The above results indicated that inhibiting the production of TMAO by intestinal microorganisms could become a potential target for prevention and treatment of CVD. Vegetarian diet is considered to be associated with the decrease of TMAO in plasma (Smits et al. 2018). The association between TMAO and CMD has been corroborated. However, although TMAO is one of the markers of gut microbiome that has been thoroughly studied, its mechanism of action in the pathogenesis of CVD needs to be further verified and explored. Whether the precursor substances of TMAO can regulate or predict the occurrence of early CVD also needs further study. Finally, it is necessary to explore the specific symbiotic microflora related to the pathway of TMA transformation, so as to reconstruct healthier microflora with less TMAO in CVD patients through dietary methods.

### BA

Bile is synthesized by the liver and consists mainly of BAs and cholesterol, which emulsifies and solubilizes lipids. Because BAs are toxic to bacterial cells, they selectively shape the microbiota profile of the human gut microbiota, which is recognized as an endocrine organ that plays a key role in host health. In addition, enzymes derived from gut bacteria can act on BAs, which may affect the composition of BA in the host intestine and circulation, thus affecting host physiology and metabolism (Molinero et al. 2019). Elucidating the mechanisms of microbiome-BAS-host interactions is a fascinating strategy for treating metabolic diseases.

The association between BA and cardiometabolic has been confirmed, but the causality between BA and cardiometabolic and the exact mechanism of the role of BA remain unclear. Meanwhile, there are significant differences in BA metabolism between humans and rodents, while most studies on BA and cardiometabolic diseases are conducted in rodents (Li and Dawson 2019). Through the determination of 136 young adults cardiometabolic risk factors and 8 kinds of plasma BA to reveal the relationship between BA level and cardiometabolic risk factors, the study showed that plasma BA level was associated with cardiometabolic and inflammatory risk factors (Osuna-Prieto et al. 2022). BA is believed to activate FXR receptor, vitamin D receptor, pregnane X receptor, and G protein–coupled receptor Gpbar1 to affect cardiometabolic diseases (Li et al. 2020a; Wang et al. 2016; Yang et al. 2021e). However, the complex relationship between BA metabolism, lipid metabolism, and FXR signal remains to be further studied (Callender et al. 2022; Wang et al. 2018a).

Although the mechanism of BA regulating CVD in cardiovascular tissues remains unclear, circulating BA levels can predict the occurrence of coronary artery disease (Chong Nguyen et al. 2021). Intriguingly, there is an association between BA signal transduction and salt-sensitive hypertension. BA may have the functions of regulating inflammation and balancing body fluids in patients with salt-sensitive hypertension. FXR and TGR5 are considered potential therapeutic targets. There is evidence that INT-767, as an agonist of FXR and TGR5, can effectively attenuate obesity and atherosclerosis in animal models (Ishimwe et al. 2022; Jadhav et al. 2018). Naringin has been proved to play an anti-atherosclerotic role by regulating BA synthesis through FXR/FGF15 signaling pathway (Wang et al. 2021b). However, intriguingly, intestinal FXR has also been proved to regulate the transcriptional activity of SMPD3 to promote the production of ceramide, thereby increasing the instability of atherosclerotic plaque (Wu et al. 2021). A study of sterol sulfate significantly improved atherosclerotic in high-fat-diet-fed Apo E2/2 mice showed changes in BA profiles in the liver, gallbladder, serum, and stool, and increased FXR expression in the liver, suggesting that FXR was one of the pathways through which BAs were involved in sterol sulfate in attenuating atherosclerosis (Ding et al. 2021). Therefore, BA and BA receptors play an important role in atherosclerosis, mediating the regulation of signaling pathways including inflammation, oxidative stress, and lipoprotein (Qi et al. 2022). Meanwhile, a prospective cohort study by Mayerhofer et al. showed a decrease in BA levels in patients with chronic HF (Mayerhofer et al. 2017). In addition, BA level also affects glucose metabolism and T2DM (Zhang et al. 2019a). Activation of TGR5, one of the important receptors of BA, has been shown to be beneficial for improving T2DM (Du et al. 2022), with significantly reduced blood glucose levels in T2DM patients treated with high-dose TGR5 agonist sdb-756050 (Hodge and Nunez 2016). However, activation of FXR is also considered to be beneficial to improving T2D (Du et al. 2022). The role of gut flora can synthesize different BAs. The specific role of signal molecules generated by the interaction between BA and intestinal flora and the role of TGR5 and FXR receptors signal in maintaining glucose homeostasis remains to be further confirmed (Gao et al. 2022).

It is significantly important to maintain the homeostasis balance between gut flora and BAs to play physiological functions in various metabolic pathways and prevent pathological progression of diseases. The complex association between BA metabolism, FXR signaling, and dyslipidemia may be a new target for intervention in CMD (Callender et al. 2022), but nevertheless, the causal mechanism between changes in BA signaling and CMD needs to be further investigated.

### Others

A series of metabolites produced by gut flora play a crucial role in human physiology, among which the three main tryptophan (Trp) metabolic pathways mediated by intestinal flora such as serotonin (5-hydroxytryptophan), kynine (Kyn), and indole derivatives have received extensive attention. Allison et al. also summarized the main ways that tryptophan, a metabolite of intestinal flora, affects diseases, especially the 5-HT, Kyn, and AhR pathways, which are affected differently in diseases, but are still closely connected (Agus et al. 2018).

In addition to tryptophan, phenylalanine and its metabolized tyrosine are also aromatic amino acids that can affect neural immunity. Tyrosine can be further converted into neurotransmitters, adrenaline, and noradrenaline (Jin et al. 2021). In patients with advanced atherosclerosis, tryptophan, indole-3-propionic acid, hippuric acid, and other phenyl-derived metabolites have changed and are associated with atherosclerosis (Cason et al. 2018). Our previous study found that L-phenylalanine, L-valine, and L-methionine were three metabolites involved in intestinal and blood metabolism in L-NAME-induced hypertensive rats, which may be related to the development mechanism of hypertension (Yuan et al. 2022). Studies have shown that phenylalanine can be metabolized by gut microbiota to produce phenylpyruvic acid (Dodd et al. 2017; Nemet et al. 2020) and finally produce polyacrylic acid. Polyacrylic acid is then circulated through the portal venous system to be metabolized in the liver to produce Phenylacetylglutamine (PAGln) (Nemet et al. 2020). A recent study also found that the key bacterial enzyme porA for PAGln synthesis increased in patients with atrial fibrillation. PAGln might participate in the pathogenesis of atrial fibrillation by promoting oxidative stress and apoptosis of atrial muscle cells (Fang et al. 2022).

Furthermore, a study has shown elevated PAGln level is an independent risk factor for HF and is associated with a higher risk of cardiovascular death (Zong et al. 2022). Meanwhile, elevated PAGln levels were also associated with acute myocardial infarction and T2DM (Tang et al. 2022). However, as a potential biomarker of cardiovascular disease, the mechanism by which PAGln acts through gut-heart needs to be verified by more cohort studies (Chen et al. 2020a).

## Potential therapeutic means

**Fig. 5:**
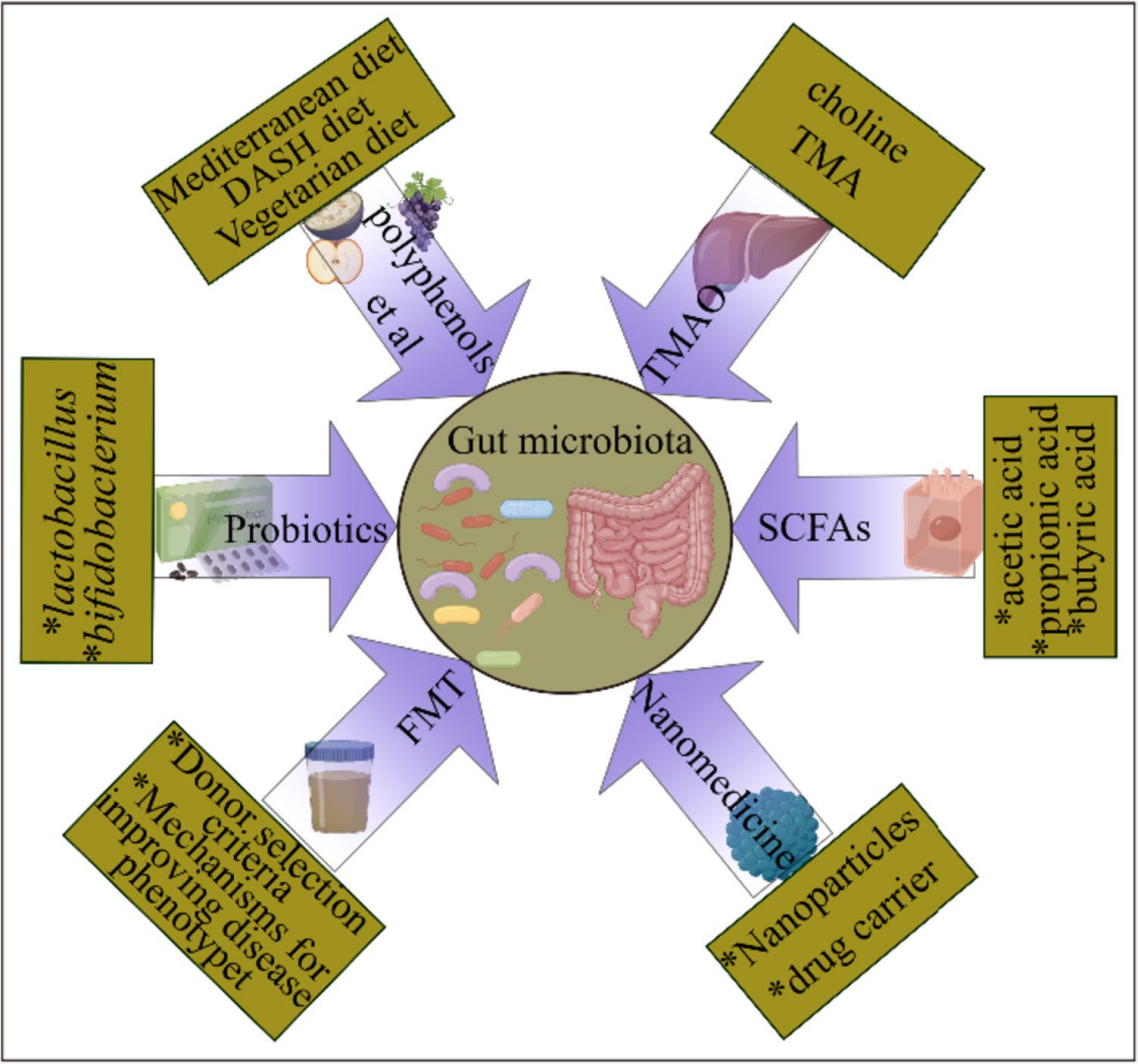
The possible application direction of targeting gut flora in the treatment of CMD. SCFA, short-chain fatty acid; TMA, trimethylamine; TMAO, trimethylamine oxide; FMT, fecal bacteria transplantation

Metabolites produced by gut flora through fermentation of indigestible fibers can be partially absorbed into the host circulation, thus acting on remote target organs. Metabolites produced by microorganisms may have synergistic effects on promoting human health. The relationship between host CMD susceptibility and changes in microbiota and metabolites makes intestinal microbiota a new target for CMD therapy. The possible directions of application of targeting the gut microbiota to treat CMD are illustrated in Fig. 5 (by Figdraw).

### Repair the impaired intestinal barrier and reduce inflammation

The apical junctional complex consisting of mucus and tight junctions between intestinal epithelial cells and luminal contents together make up the intestinal barrier (Lewis and Taylor 2020; Yang et al. 2021c). Commensal bacteria are present in the mucus layer of the intestinal lumen. As a result of mucin cleavage, the loose mucus expands in volume, allowing bacteria to penetrate the mucus. Gut microbiota and their metabolites can affect the host’s neuroimmune regulation after entering the circulation system through intestinal leakage (Yousefi et al. 2022). After the occurrence of intestinal leakage, the thickness of mucus in the luminal decreases, leading to the entry of intestinal bacteria and antigens into the circulation, thereby triggering systemic inflammatory pathways (Feng et al. 2019; Ma and Li 2018), which increases the risk of cardiovascular diseases (CVD) (Manolis et al. 2022). Adherence to the Mediterranean diet is associated with fewer adverse cardiovascular events and lower circulating LPS levels (Pastori et al. 2017). Dietary bioactive molecules synthesized and metabolized by gut microbes can affect hypertension after entering the circulation system (Cookson 2021).

Brandsma et al. believed that identified pattern recognition receptors and antimicrobial peptides as key factors controlling the composition of the gut microbiota, and defects in these genes lead to leakage of endotoxin into the circulation, resulting in low-grade chronic inflammation and insulin resistance (Brandsma et al. 2015). T2D metabolic inflammation is also known to be associated with increased intestinal permeability and gut-derived lipopolysaccharide translocation. A number of other natural foods have also been proposed to repair the intestinal barrier, reduce intestinal inflammation, and inhibit intestinal metabolic disorders (Hu et al. 2021; Yang et al. 2021d; Yuan et al. 2021). For example, Berberine, which improves intestinal barrier function and reduces inflammation caused by intestinal microbiota-derived LPS, is considered to have the potential to treat atherosclerotic cardiovascular disease and metabolic diseases (Yang et al. 2021c). In conclusion, prevention of the occurrence of inflammation, increased intestinal permeability, and damaged intestinal barrier caused by intestinal leakage may be potential means to intervene in CMD.

### Targeted regulation of gut microbiota

#### Dietary patterns can regulate gut microbiota to prevent CMD

The gut flora catabolisms the unused dietary components of the host, which is associated with the multiple metabolisms of central carbon and amino acids and the biosynthesis of secondary metabolites (Griffin et al. 2015; Xu and Yang 2021). The metabonomic evidence showed that the metabolites of gut flora affected the host physiology (Xu and Yang 2020), and regulated the level of these metabolites in the circulation may help prevent the occurrence of CMD (Griffin et al. 2015; Xu and Yang 2020). Dietary polysaccharides, flavonoids, polyphenols, and other compounds can regulate gut microbiota and affect the production of gut microbiota–derived metabolites (Feng et al. 2022), and constraint-based modeling analysis is considered to be a promising method to study the interaction between diet and gut microbiota (Blasco et al. 2021).

A large body of research have shown that dietary polyphenols have beneficial effects in improving hypertension and hyperlipidemia and promoting the regeneration of vascular endothelial cells (Szczepańska et al. 2022). In addition, coenzyme Q10, which is rich in foods such as soybeans and nuts, has been proved to have the function of protecting the heart and blood vessels, and the mechanisms by which play a cardioprotective role include increasing ATP production in myocardial cells, antioxidation, improving fat content and improving endothelial function (Gutierrez-Mariscal et al. 2021; Testai et al. 2021). Sterols from plant diet, such as sitosterol, have the effect of antioxidation and improving blood lipids (Babu and Jayaraman 2020; Szczepańska et al. 2022). Other food components beneficial to CMD include Omega-3 and Vitamin E, which can reduce platelet aggregation and reduce the risk of CVD (Cao and Weaver 2022; Innes and Calder 2020; Margina et al. 2020).

The previous studies argued that the dietary patterns that were conducive to CMD and lowered the risk of death from CVD include Mediterranean diet (Martínez-González et al. 2019; Rees et al. 2019; Salas-Salvadó et al. 2018), the DASH diet (Chiavaroli et al. 2019; Filippou et al. 2020; Juraschek et al. 2020; Liu et al. 2021a), and Vegetarian diets (Chiu et al. 2020; Quek et al. 2021; Trautwein and McKay 2020). As in the Wang et al. study, adherence to the Mediterranean diet would have a significant impact on the gut microbiota related to SCFA production, secondary BA production and degradation of plant-derived polysaccharides, and the lack of *P. cpori* in the gut microbiota of a group of participants, which may be the reason for their risk protection of heart metabolic diseases (Wang et al. 2021a). Due to the heterogeneity of individuals, different dietary patterns will affect diet-related individual metabolic patterns; however, the relationship between individual metabolic characteristics and CMD has been confirmed. The characteristics of dietary-derived metabolites are generally important in CMD. Ravi V. Shah et al. found that food components/catabolites (such as fish and long-chain unsaturated triacylglycerol) interacted with host characteristics (e.g., intestinal flora) and involved in CMD- and CVD- related pathways (e.g., ceramide/sphingomyelin lipid metabolism) (Shah et al. 2022). Therefore, more population cohorts need to be designed, and personalized diet should be designed based on individual metabolic characteristics.

#### Probiotics

Probiotics, known as the next generation of viable bacteriological drugs, change the composition and metabolites of gut flora through colonization in the gut, which is closely related to human health, especially the protective effect on cardiometabolic diseases such as hypertension, atherosclerosis, and insulin resistance (Chi et al. 2020; Leustean et al. 2018; O'Morain and Ramji 2020). In recent years, a large body of researches have accumulated in vivo experimental data of probiotics to lower BP. Several meta-analysis studies have shown that probiotics may lower BP, which is expected to provide a new method for the treatment of hypertension (Chi et al. 2020; Lewis-Mikhael et al. 2020; Liang et al.; Qi et al. 2020). In a meta-analysis of data from 11 randomized controlled trials (RCTs), probiotic supplementation lowered BP in patients with T2DM (Hendijani and Akbari 2018). However, the data were not entirely consistent; Dong et al.’s meta-analysis of 18 trials in patients with MS showed no significant changes in BP caused by probiotic supplementation (Dong et al. 2019). The research of probiotic intervention in diabetes is also one of the ongoing hot topics. A meta-analysis of 13 RCTs found that supplementation with probiotics reduced FBG levels but did not change HbA1c levels (Ardeshirlarijani et al. 2019). The study of Liang et al. (2020) was similar, especially the mixed probiotics had a great effect on the level of FBG but had no effect on the level of HbA1c and insulin (Liang et al.). However, two meta-analyses showed that probiotics did not have any significant effects on markers of glucose metabolism and insulin function (Barengolts et al. 2019; Dong et al. 2019). Multiple meta-analyses evaluating the potential of probiotics to regulate lipid levels have shown that probiotic supplements help improve dyslipidemia (Companys et al. 2020; Hadi et al. 2020; Hendijani and Akbari 2018; Liang et al.; Yan et al. 2019). In addition, probiotics also regulate metabolites in the gut that are associated with cardiovascular disease. Liang et al. used Lactis F1-3–2 intervention to regulate TMA-TMAO in cecum and serum, improve lipid metabolism, and attenuate TMAO-induced atherosclerosis (Liang et al. 2020). In conclusion, probiotics have the effect of regulating blood glucose, BP, and blood lipids, which is beneficial to improve CMD such as hypertension, diabetes, and atherosclerosis. However, other studies have shown that probiotics supplementation did not significantly improve the levels of CMD-related markers. Therefore, further population-based studies with larger cohorts are needed to verify the effect of probiotics on improving CMD by removing the influencing factors of other diseases except CMD. Nevertheless, probiotics still show great potential in the intervention of CMD. Moreover, the evaluation of the safety and genetic stability of probiotics is also significantly important. After clinical and evidence-based medical evaluation, probiotics are expected to become oral microbial drugs in vivo.

### Improve the metabolism of gut microbiota

#### Dietary intervention regulates SCFAs

It is of particular importance to improve chronic metabolic diseases by regulating intestinal microbial function and metabolites through dietary intervention (Wu et al. 2011). More fiber intake is believed to produce more SCFAs. Animal-based diet and plant-based diet would affect the production of gut flora and gut flora metabolite SCFAs. Animal-based diet could significantly reduce the concentration of acetate and butyrate in the feces of the host (David et al. 2014).

Numerous studies have implicated the association between gut flora, diet, and health, and different diets can affect human health and CMD risk. The Mediterranean diet, for example, emphasizes a higher intake of vegetable and grains, which is associated with higher SCFA levels and gut flora richness (Garcia-Mantrana et al. 2018). Dietary fiber promoted the increase of SCFA-producing strains, increased species diversity and richness, improved glycosylated hemoglobin, and decreased production of harmful compounds such as indoles and hydrogen sulfide in T2DM patients (Zhao et al. 2018). The diet with low dietary fiber would not only reduce the production of SCFA, but also increased the production of metabolites harmful to mucin. A study showed that dietary fiber deprivation might be the cause of damage to the colonic mucus barrier (Desai et al. 2016). Low prebiotic fiber diet may lead to CMD. David et al. showed that the possible causes of hypertension due to the deficiency of prebiotic fibers include the formation of hypertensinogenic intestinal flora and the reduction of SCFA production (Kaye et al. 2020).

Intervention with CMD based on microbial SCFA diet may have good effect, high safety, and high compliance. However, due to the huge heterogeneity of individual gut flora, the metabolites produced by it may act on multiple tissues and targets (Turnbaugh 2020). Therefore, it is necessary to establish personalized diet strategies and conduct more population experiments to verify its effects.

#### Dietary and pharmacological interventions targeting TMAO levels

Diets rich in TMAO precursors, such as red meat and eggs, and consumption of animal protein and saturated fats may increase the amount of circulating TMAO. The intake of choline and carnitine in the diet can affect the level of circulating TMAO. Compared with omnivores, vegetarians have lower levels of circulating TMAO (Koeth et al. 2013). However, a predominantly red meat–based diet would increase the concentration of TMAO in the circulation, and changing the red meat–based diet to a white meat non meat protein diet could reduce the circulating TMAO concentration in a few weeks (Wang et al. 2019). The increase of circulating TMAO level by high-fat diet may be associated with the increase of oxygen and nitrate utilization after damaging the oxygen uptake of mitochondria to host intestinal epithelial cells (Yoo et al. 2021).

Therefore, it is possible to reduce the intake of TMAO from foods in the diet, such as choosing plant proteins as sources of protein or supplementing the Mediterranean diet, to promote cardiovascular health and reduce the incidence of cardiovascular events (Estruch et al. 2018; Mei et al. 2021; Yang et al. 2021b). It is an urgent problem to develop an economical and effective diet to reduce TMAO and attenuate CMD, which has potential significant public health benefits, among which plant-based substances also deserve attention (Iglesias-Carres et al. 2021).

### Other techniques targeting gut microbiota to improve CMD

#### FMT

FMT has received widespread attention worldwide as an ecological therapy to restore the patient’s gut microbiota. A large body of preclinical and clinical studies have shown that FWT can be used in the treatment of CMD (Zheng et al. 2022). FMT can be used for the treatment of T1D and T2D. Studies have shown that β cell function is restored in T1D patients after FMT (Zhang et al. 2019b) Another study also showed that patients with T2D could maintain normal metabolism after diet-induced weight loss after autologous FMT (Chang et al. 2015). Previous research by our team has found that Washed Microbiota Transplantation (WMT) could also lower BP in patients with hypertension, especially in those who did not take antihypertensive drugs (Zhong et al. 2021). Moreover, a retrospective analysis of the improvement of blood glucose after WMT treatment for various reasons in the First Hospital of Guangzhou Pharmaceutical University from December 2016 to April 2022 found that WMT also significantly improved FBG (Wu et al. 2022a). Interestingly, the recent study from our group, which included 237 patients (42 with MS and 195 without MS), found that WMT treatment significantly restored gut microbiota homeostasis and improved blood glucose, lipid profile, BP, and BMI in patients with MS (Wu et al. 2022b). Apart from that, FMT is also making continuous attempts and breakthroughs in the treatment of tumor, nerve, and autoimmune diseases (Opoku-Acheampong et al. 2022). Most studies have shown that the transplantation of microorganisms into the gut of patients with CMD results in changes in the disease phenotype. The microbiota of the disease donor would replicate in the mouse gut. However, there were also cases in which FMT failed to develop a pathological phenotype in the recipient animals (Walter et al. 2020). In addition, probiotics that have been evaluated by clinical and evidence-based medicine may be added to the donor stool for personalized reinforcement of the donor microbiota based on patient characteristics. FMT has been fully recognized for the treatment of diseases, especially digestive tract diseases, but its role in the treatment of CMD is still controversial. In particular, the application of FMT is limited by the selection of donors and the heterogeneity of donor feces (Zheng et al. 2022). More clinical trial data are needed to promote the progress of FMT technology. It is necessary to optimize the design and develop more standardized application guidelines in the future.

#### Microbiota and nanomedicine-based approaches

Nanomedicine has been widely used in the diagnosis and targeted therapy of cancer, CMD, and other diseases, especially in the delivery of drugs as a carrier and the improvement of pharmacokinetics. Nanotechnology can promote the delivery of microbes and their lead compounds for targeted therapy of gut microbiota, protein profiling, and cholesterol modulation such as clearance of low-density lipoprotein cholesterol, thus promoting the prevention and treatment of CMD (Kazemian et al. 2020). Nanotechnology can be applied to modulate immunity and alleviate inflammation (Gan et al. 2018). Wang et al. applied broad-spectrum ROS-scavenging nanoparticles to reduce oxidative stress and thereby alleviate atherosclerosis (Wang et al. 2018b). Nanotechnology based on extracellular vesicles has brought opportunities for the development of nanomedicine. Exosomes and outer membrane vesicles regulate intestinal immunity and homeostasis by participating in the interaction between intestinal epithelial cells, immune cells, and gut flora (Li et al. 2022b). Intestinal bacteria and metabolites can change the physicochemical properties of nanoparticles, and nanoparticles can regulate bacterial metabolism and communication with intestinal lymphoid tissues (Javed et al. 2020). Based on the interaction between nanoparticles and intestinal flora, and the application of nanotechnology in the delivery and release of probiotics, it has the potential to apply it to target gut flora for the treatment of CMD. However, more biological studies are needed regarding the clinical application of nanotechnology-based particulate products (Mahmoudi 2018). As for nanomaterial as an emerging interdisciplinary discipline offers fascinating prospects for the treatment of CMD. However, the association between gut flora, nanomedicine, and CMD needs more clinical and cohort studies.

## Conclusions and future perspectives

The gut microbiome profile of CMD has been initially described and is characterized by decreased gut microbiota abundance, absence of SCFA-producing bacteria, and abnormal trimethylamine metabolism, which are more closely related to metabolic disorders. A precise description and correlation study of CMD phenotype and intestinal physiological function were carried out, and combined with causal studies. Personalized microbiome therapy may be the most promising direction, which may require targeted capture of functional strains or genes that affect the progression of CMD and the physiological function of the host gut. The association of gut microbiota changes with distal target organs is also of interest, including the gut-heart, gut-vascular, gut-liver, and gut-brain axes. In addition, enterovirus studies, especially bacteriophages, have also been considered to have the potential to target the regulation of the gut microbiome due to their specificity (Hsu et al. 2019; Shkoporov and Hill 2019). Combined enterovirus and whole genome sequencing results from 196 patients with MS (a highly prevalent clinical condition preceding cardiometabolic disease) showed that the enterovirus was associated with a highly widely distributed intestinal phage family, named Candidatus Heliusviridae (de Jonge et al. 2022).

Challenges remain in fully integrating gut microbiota into clinical design. The variation factors of gut flora are very complex. The results of increased opportunistic pathogens in the gut of CMD patients are not completely reproducible, which may be related to the large individual differences. There is also no uniform standard for fecal sample collection for gut microbiota research, and different intestinal segments and different collection methods will contain different microbiota. In order to solve the above problems, it is necessary to turn the correlation study to the causal mechanism study, explore the exact downstream molecular pathway, carry out a large-scale prospective cohort study based on the population, and clinical and rodent correlation study to reveal the complex mechanism between gut flora and CMD. However, the factors driving causality may be diverse, including the host intestinal environment, intestinal transit time, and PH changes. In addition, we can also consider the method of gene editing to tap fluorescent proteins into bacteria or find specific molecular targets of bacteria for visual tracking, which is helpful to explore the pathway and target of the role of bacteria.

The small intestine plays a key role in digestion and nutrient absorption, and bacteria have a great impact on metabolism, intestinal barrier, immune response, etc. However, the overall stable changes of intestinal flora caused by CMD phenotype may also occur at time intervals, and the changes in different intestinal segments may not be consistent. Therefore, we propose that nanotechnology may be a method for gut microbiome research. Although the causal mechanism between gut microbiome and CMD is still unclear, there has been substantial evidence that the changes of gut microbiota may be the cause of CMD; in particular, the application of germ-free mice and FMT have brought us numerous animal research and clinical research data. In order to fully elucidate the specific mechanism of intestinal microbiome mediating CMD, and to guide the therapeutic intervention of CMD via probiotics, precision nutrition targeting intestinal metabolites, FMT, and advanced nanomedicine technology, thus we must elucidate the physiological factors that jointly affect the microbiome and disease, delineate the personalized microbiome profile, and capture a holistic view of pathogenic mechanisms.

## Supplementary Information

Below is the link to the electronic supplementary material.

Supplementary file1 (PDF 170 KB)

## Data Availability

The authors declare that all of the data and the material used in this study are available within this article. All data generated or analyzed in this study can be obtained from the authors upon reasonable request.
