# Supplementary material for: Therapeutic applications of gut microbes in cardiometabolic diseases: current state and perspectives
Source: Appl Microbiol Biotechnol. 2024 Jan 20;108:156. doi: 10.1007/s00253-024-13007-7 (PMC10799778; doi:10.1007/s00253-024-13007-7)
Supplement: Supplementary file 1 [file 253_2024_13007_MOESM1_ESM.pdf]

APPLIED MICROBIOLOGY AND BIOTECHNOLOGY

Therapeutic applications of gut microbes in cardiometabolic diseases: current state and perspectives

Lin Yuan [a,b], Ying Li [b], Moutong Chen [b], Liang Xue [b], Juan Wang [d], Yu Ding [c], Qihui Gu[b], Jumei Zhang [b], Hui Zhao [b], Xinqiang Xie [b,*] and Qingping Wu [b,*]

[a] *School of Food and Biological Engineering, Shaanxi University of Science and Technology, Xi'an 710021, China; gdim_yuanlin@163.com (L.Y.)*

[b] *Guangdong Provincial Key Laboratory of Microbial Safety and Health, State Key Laboratory of Applied Microbiology Southern China, Institute of Microbiology, Key Laboratory of Agricultural Microbiomics and Precision Application, Ministry of Agriculture and Rural Affairs, Guangdong Academy of Sciences, Guangzhou 510070, China;*

[c] *Department of Food Science and Engineering, Institute of Food Safety and Nutrition, College of Science & Engineering, Jinan University, Guangzhou 510632, China;*

[d] *College of Food Science, South China Agricultural University, Guangzhou 510642, China;*

* Correspondence: woshixinqiang@126.com (X.X.); wuqp203@163.com (Q.W.)

Affiliation: Institute of Microbiology, Guangdong Academy of Sciences.

No. 100, Xianlie Zhong Road, Yuexiu District, Guangzhou 510070, P. R. China

Tel/Fax: +86-20-87688132

Email: wuqp203@163.com

Table S1. Altered gut microbial compositions associated with hypertension

Table S2 Altered Intestinal Microbiota Composition Associated with Atherosclerosis

Table S1. Altered gut microbial compositions associated with hypertension

First Author (Ref. #), Year	Sample	Technique	Increase in hypertension	Decrease in hypertension
Mell et al. [1], 2015	Dahl salt-resistant rats	16S	Plasma acetate and heptanoate	S24-7, *Veillonellaceae*
Gomez-Arango et al. [2], 2016	obese pregnant women	16S		*Butyrate-producing bacteria Odoribacter*
Jackson et al. [3], 2019	756 hypertensive patients, 1790 controls	16S	*Lactobacillaceae, Streptococcaceae*	*Dehalobacteriaceae, Christensenellaceae, Oxalobacteraceae, Mollicutes, Rikenellaceae, Clostridia, Anaeroplasmataceae, Peptococcaceae*
Huart et al. [4], 2019	38 hypertensive patients, 7 pre-hypertensive patients, 9 controls	16S	*Clostridum sensu stricto*	*Ruminococcaceae, Clostridiales*
Verhaar et al. [5], 2020	4672 subjects	16S	*Streptococcus*	*Roseburia, Clostridium sensu stricto, Roseburia hominis, Romboutsia, Ruminococcaceae, Enterorhabdus*
Iñaki Robles et al.[6], 2021	8 DOCA-salt rats	16S	*lactate-producing bacteria*	*Oscillospira*
Larissa et al. [7], 2021	hypertensive rats induced by high-fat diet	16S	*Lactobacillales, Clostridiales, Enterobacteriales*	*Bacteroidales, Desulfovibrionales, Methanobacteriales, Gastranaerophilales, Selenomonadales.*
Chao-Yue et al. [8], 2021	Spontaneously hypertensive rats	16S	*the ratio between Firmicutes and Bacteroidetes*	

Haicui et al. [9], 2022	hypertensive rats	16S	*Proteobacteria*	*Bacteroidales*_S24-7
Georgianna et al. [10], 2022	hypertensive rats induced by high-fat diet	16S	*Lachnospiraceae, Clostridiaceae 1, Peptostreptococcaceae, Enterobacteriaceae*	*Ruminococcaceae, Methanobacteriaceae, Muribaculaceae*
Lin et al.[11], 2022	L-NAME-induced hypertensive rats	16S	*Christensenellaceae, Streptococcaceae,* UUG-010	

Table S2 Altered Intestinal Microbiota Composition Associated with Atherosclerosis and coronary artery disease

First Author (Ref. #), Year	Sample	Technique	Increase in CVD	Decrease in CVD
Yin et al.[12], 2015	141 stroke/TIA patients vs. 94 asymptomatic control subjects	16S rRNA	*Enterobacteriaceae* *Proteobacteria* *Escherichia/Shigella*	*Bacteriodetes* *Bacteriodales* *Bacteroidaceae* *Bacteriodes*
Feng et al. [13], 2016	59 CAD patients vs. 43 healthy control subjects	Fecal metagenome	*Streptococcus sp.* M334 *and* M143 *Clostridium sp.* HGF2	
Jie et al.[14], 2017	218 ACVD and 187 healthy controls.	Fecal Metagenomic	*Streptococcus and Escherichia*	*Bacteroides and Prevotella*
Kasahara et al.[15],2018	Atherosclerotic murine model	16S sequencing		*Roseburia*
Zhiyang et al. [16], 2021	Atherosclerotic mice model	16S sequencing	*Helicobacter spp.*	
Xiao-Lin et al. [17], 2022	Atherosclerotic mice model	16S sequencing	*Firmicutes*	*Bacteroidetes*

Reference

[1] B. Mell, V.R. Jala, A.V. Mathew, J. Byun, H. Waghulde, Y. Zhang, B. Haribabu, M. Vijay-Kumar, S. Pennathur, B. Joe, Evidence for a link between gut microbiota and hypertension in the Dahl rat, Physiological genomics 47(6) (2015) 187-197.
[2] L.F. Gomez-Arango, H.L. Barrett, H.D. McIntyre, L.K. Callaway, M. Morrison, M.D. Nitert, S.T. Grp, Increased Systolic and Diastolic Blood Pressure Is Associated With Altered Gut Microbiota Composition and Butyrate Production in Early Pregnancy, Hypertension 68(4) (2016) 974-981.
[3] M.A. Jackson, S. Verdi, M.-E. Maxan, C.M. Shin, J. Zierer, R.C. Bowyer, T. Martin, F.M. Williams, C. Menni, J.T. Bell, Gut microbiota associations with common diseases and prescription medications in a population-based cohort, Nature communications 9(1) (2018) 1-8.
[4] J. Huart, J. Leenders, B. Taminiau, J. Descy, A. Saint-Remy, G. Daube, J.-M. Krzesinski, P. Melin, P. de Tullio, F. Jouret, Gut Microbiota and Fecal Levels of Short-Chain Fatty Acids Differ Upon 24-Hour Blood Pressure Levels in Men, Hypertension 74(4) (2019) 1005-1013.
[5] B.J. Verhaar, D. Collard, A. Prodan, J.H. Levels, A.H. Zwinderman, F. Bäckhed, L. Vogt, M.J. Peters, M. Muller, M. Nieuwdorp, Associations between gut microbiota, faecal short-chain fatty acids, and blood pressure across ethnic groups: the HELIUS study, European heart journal 41(44) (2020) 4259-4267.
[6] I. Robles-Vera, N. de la Visitación, M. Toral, M. Sánchez, M. Romero, M. Gómez-Guzmán, F. Vargas, J. Duarte, R. Jiménez, Changes in Gut Microbiota Induced by Doxycycline Influence in Vascular Function and Development of Hypertension in DOCA-Salt Rats, Nutrients 13(9) (2021) 2971.
[7] L.R. Brandão, J.L. de Brito Alves, W.K.A. da Costa, G.d.A.H. Ferreira, M.P. de Oliveira, A.G. da Cruz, V. de Andrade Braga, J. de Souza Aquino, H. Vidal, M.F. Noronha, Live and ultrasound-inactivated Lacticaseibacillus casei modulate the intestinal microbiota and improve biochemical and cardiovascular parameters in male rats fed a high-fat diet, Food Function 12(12) (2021) 5287-5300.
[8] C.-Y. Kong, Z.-M. Li, Y.-Q. Mao, H.-L. Chen, W. Hu, B. Han, L.-S. Wang, Probiotic yogurt blunts the increase of blood pressure in spontaneously hypertensive rats via remodeling of the gut microbiota, Food Function 12(20) (2021) 9773-9783.
[9] H. Wu, L. Jiang, T.-F. Shum, J. Chiou, Elucidation of anti-hypertensive mechanism by a novel Lactobacillus rhamnosus AC1 fermented soymilk in the deoxycorticosterone acetate-salt hypertensive rats, Nutrient 14(15) (2022) 3174.
[10] G. de Araújo Henriques Ferreira, M. Magnani, L. Cabral, L.R. Brandão, M.F. Noronha, J. de Campos Cruz, E.L. de Souza, J.L. de Brito Alves, Potentially probiotic limosilactobacillus fermentum Fruit-Derived strains alleviate cardiometabolic disorders and gut microbiota impairment in male rats fed a high-Fat diet, Probiotics Antimicrobial Proteins 14(2) (2022) 349-359.
[11] L. Yuan, Y. Li, M. Chen, L. Xue, J. Wang, Y. Ding, J. Zhang, S. Wu, Q. Ye, S. Zhang, R. Yang, H. Zhao, L. Wu, T. Liang, X. Xie, Q. Wu, Antihypertensive Activity

of Milk Fermented by *Lactiplantibacillus plantarum* SR37-3 and SR61-2 in L-NAME-Induced Hypertensive Rats, Foods 11(15) (2022).
[12] J. Yin, S.X. Liao, Y. He, S. Wang, G.H. Xia, F.T. Liu, J.J. Zhu, C. You, Q. Chen, L. Zhou, Dysbiosis of gut microbiota with reduced trimethylamine-N-oxide level in patients with large-artery atherosclerotic stroke or transient ischemic attack, Journal of the American Heart Association 4(11) (2015) e002699.
[13] Q. Feng, Z. Liu, S. Zhong, R. Li, H. Xia, Z. Jie, B. Wen, X. Chen, W. Yan, Y. Fan, Integrated metabolomics and metagenomics analysis of plasma and urine identified microbial metabolites associated with coronary heart disease, Scientific reports 6(1) (2016) 1-14.
[14] Z. Jie, H. Xia, S.-L. Zhong, Q. Feng, S. Li, S. Liang, H. Zhong, Z. Liu, Y. Gao, H. Zhao, The gut microbiome in atherosclerotic cardiovascular disease, Nature communications 8(1) (2017) 1-12.
[15] K. Kasahara, K.A. Krautkramer, E. Org, K.A. Romano, R.L. Kerby, E.I. Vivas, M. Mehrabian, J.M. Denu, F. Bäckhed, A.J. Lusis, Interactions between Roseburia intestinalis and diet modulate atherogenesis in a murine model, Nature microbiology 3(12) (2018) 1461-1471.
[16] Z. Lv, X. Shan, Q. Tu, J. Wang, J. Chen, Y. Yang, Ginkgolide B treatment regulated intestinal flora to improve high-fat diet induced atherosclerosis in ApoE−/− mice, Biomedicine Pharmacotherapy 134 (2021) 111100.
[17] X.L. Li, J.J. Cui, W.S. Zheng, J.L. Zhang, R. Li, X.L. Ma, M. Lin, H.H. Guo, C. Li, X.Y. Yu, Bicyclol Alleviates Atherosclerosis by Manipulating Gut Microbiota, Small 18(9) (2022) 2105021.
